# *Mycobacterium tuberculosis* Lineage Influences Innate Immune Response and Virulence and Is Associated with Distinct Cell Envelope Lipid Profiles

**DOI:** 10.1371/journal.pone.0023870

**Published:** 2011-09-08

**Authors:** Nitya Krishnan, Wladimir Malaga, Patricia Constant, Maxine Caws, Tran Thi Hoang Chau, Jenifer Salmons, Nguyen Thi Ngoc Lan, Nguyen Duc Bang, Mamadou Daffé, Douglas B. Young, Brian D. Robertson, Christophe Guilhot, Guy E. Thwaites

**Affiliations:** 1 Centre for Molecular Microbiology and Infection, Imperial College London, London, United Kingdom; 2 CNRS, IPBS (Institut de Pharmacologie et de Biologie Structurale), Toulouse, France; 3 Université de Toulouse, UPS, IPBS, Toulouse, France; 4 Oxford University Clinical Research Unit, Ho Chi Minh City, Vietnam; 5 Hospital for Tropical Diseases, Ho Chi Minh City, Vietnam; 6 Pham Ngoc Thach Hospital for Tuberculosis and Lung Disease, Ho Chi Minh City, Vietnam; 7 National Institute for Medical Research, London, United Kingdom; Institut Pasteur, France

## Abstract

The six major genetic lineages of *Mycobacterium tuberculosis* are strongly associated with specific geographical regions, but their relevance to bacterial virulence and the clinical consequences of infection are unclear. Previously, we found that in Vietnam, East Asian/Beijing and Indo-Oceanic strains were significantly more likely to cause disseminated tuberculosis with meningitis than those from the Euro-American lineage. To investigate this observation we characterised 7 East Asian/Beijing, 5 Indo-Oceanic and 6 Euro-American Vietnamese strains in bone-marrow-derived macrophages, dendritic cells and mice. East Asian/Beijing and Indo-Oceanic strains induced significantly more TNF-α and IL-1β from macrophages than the Euro-American strains, and East Asian/Beijing strains were detectable earlier in the blood of infected mice and grew faster in the lungs. We hypothesised that these differences were induced by lineage-specific variation in cell envelope lipids. Whole lipid extracts from East Asian/Beijing and Indo-Oceanic strains induced higher concentrations of TNF-α from macrophages than Euro-American lipids. The lipid extracts were fractionated and compared by thin layer chromatography to reveal a distinct pattern of lineage-associated profiles. A phthiotriol dimycocerosate was exclusively produced by East Asian/Beijing strains, but not the phenolic glycolipid previously associated with the hyper-virulent phenotype of some isolates of this lineage. All Indo-Oceanic strains produced a unique unidentified lipid, shown to be a phenolphthiocerol dimycocerosate dependent upon an intact *pks15/1* for its production. This was described by Goren as the ‘attenuation indictor lipid’ more than 40 years ago, due to its association with less virulent strains from southern India. Mutation of *pks15/1* in a representative Indo-Oceanic strain prevented phenolphthiocerol dimycocerosate synthesis, but did not alter macrophage cytokine induction. Our findings suggest that the early interactions between *M. tuberculosis* and host are determined by the lineage of the infecting strain; but we were unable to show these differences are driven by lineage-specific cell-surface expressed lipids.

## Introduction

**Figure 1:**
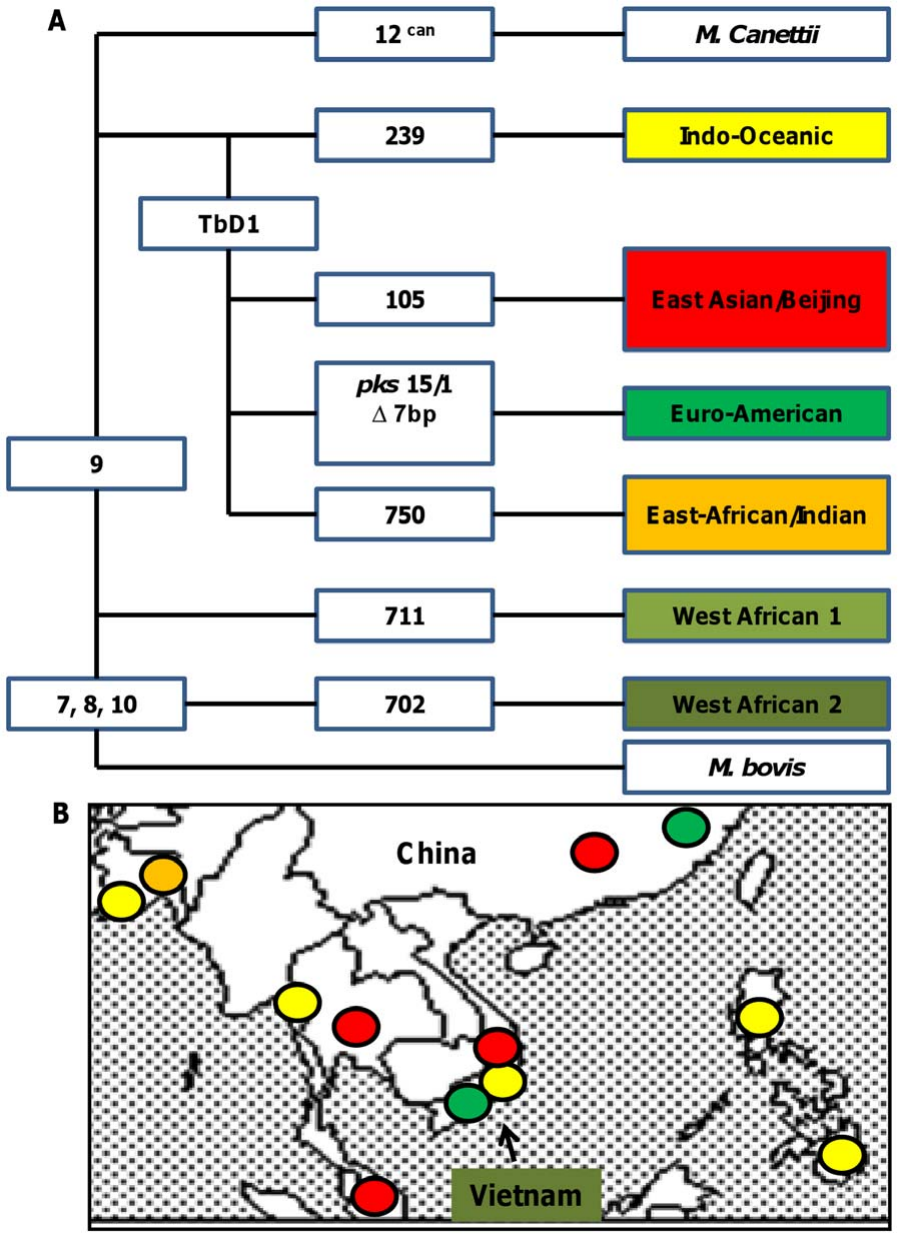
The phylogeny of *M. tuberculosis* in South East Asia. Large sequence polymorphisms define six major lineages of *M. tuberculosis* (A) which are strongly associated with specific geographical regions (B). In Vietnam, three lineages cause the majority of disease: the East Asian/Beijing, the Indo-Oceanic, and the Euro-American (adapted from reference 1). Numercial values in the figure represent regions of deletions (RD) that define each of the lineages.

Large chromosomal deletions, also termed large sequence polymorphisms, classify *Mycobacterium tuberculosis* into six major lineages, each strongly associated with specific geographical human population [1] (**figure 1**). Whether bacterial lineage influences the development of tuberculosis disease is uncertain, although there is long-standing evidence that some strains of *M. tuberculosis* are more virulent than others [2]. Fifty years ago Mitchison and colleagues reported strains of *M. tuberculosis* from southern India were less virulent in guinea pigs than UK strains [3] and, more recently, others have documented a wide variation in the virulence of *M. tuberculosis* strains following infection of mice [4], [5], [6], [7] and rabbits [8], [9]. The significance of these findings to human disease, however, remains uncertain.

Perhaps the most compelling evidence for clinically important *M. tuberculosis* genetic variation has derived from the study of tuberculosis outbreaks and has suggested the strains responsible cause disease through subversion of the host innate immune response [2]. For example, strain HN878 (a member of the East Asian/Beijing lineage), which caused several disease outbreaks in Texas, USA, was found to be hyper-virulent in animal models [10]. Its unusual virulence was attributed to the production of a cell wall phenolic glycolipid (PGL), which suppressed the release of tumour necrosis factor-alpha (TNF-α) and interleukin (IL) -12p40 from human monocyte-derived macrophages compared with the laboratory strain H37Rv [11]. Subsequently, the effect of PGL was found to be influenced by the background of the strain. *M. tuberculosis* strain, H37Rv, expressing PGL suppressed pro-inflammatory cytokine production from human monocytes, *in vitro*, but did not display increased virulence in mice or guinea pigs [12]. Similarly, a strain responsible for an outbreak of tuberculosis amongst school children in the UK (and a member of the African-Indian lineage) was also associated with an anti-inflammatory phenotype, causing suppression of IL-12p40 and up-regulation of IL-10 from macrophages [13].

It is uncertain whether these phenotypes, and the mechanisms that drive them, are strain-specific or common to the broader lineages of *M. tuberculosis* to which they belong. A recent study of a panel of 26 global clinical isolates, with representatives from all six lineages, found considerable heterogeneity in the inflammatory response induced from human macrophages by the strains. However, strains from the ‘modern’ lineages (Euro-American, East Asian/Bejing and Indian/East African) induced less inflammatory response than those from ‘ancient’ lineages (Indo-Oceanic and West African) [14]. It is hypothesised that a reduced inflammatory response may confer a selective advantage to strains from the ‘modern’ lineages, resulting in impaired bacterial control by the host, more rapidly progressive disease, and enhanced transmission.

Previously, to investigate whether some lineages of *M. tuberculosis* may be more capable than others at causing disseminated tuberculosis with meningitis we performed a case-control study in southern Vietnam, comparing 187 *M. tuberculosis* strains isolated from the cerebrospinal fluid of adults with tuberculous meningitis with 237 strains isolated from the sputum of adults with uncomplicated pulmonary tuberculosis [15]. Euro-American lineage strains were significantly less likely to cause tuberculous meningitis than strains from the Indo-Oceanic or East Asian/Beijing lineage (odds ratio 0.34, 95% confidence intervals (C.I.) 0.19–0.81, p = 0.009).

Here, we attempt to understand the basis for these observations by characterising representative strains from each lineage in macrophages, dendritic cells, and mice. We show the East Asian/Beijing and Indo-Oceanic strains induced significantly more TNF-α and IL-1β from macrophages and the East Asian/Beijing strains disseminated more rapidly to blood and spleen in mice, compared to the Euro-American strains. We hypothesise that the different phenotypes observed in these models are induced by lineage-specific differences in bacterial cell envelope lipids and we demonstrate a highly conserved lipid profile within each lineage.

## Results

First, we selected 18 strains of *M. tuberculosis* from the 424 included in the original case control study: 7 East Asian/Beijing, 5 Indo-Oceanic, and 6 Euro-American, defined by large sequence polymorphisms [16]. Strains were chosen to reflect the genotypic diversity found within each lineage in the study, subdivided by spoligotype and 12-loci mycobacterial interspersed repetitive units (MIRU) type (table 1) [15], [17], [18], [19]. Due to biosafety considerations, only isolates fully susceptible to first-line drugs were included. Representative isolates of the major spoligotypes within each of the three lineages were chosen in duplicate or triplicate, if available. All of the isolates, except one (no. 639, Euro-American), were cultured from the CSF of patients with meningitis in order to overcome any selection bias/behavioural difference in strains isolated from different sites.

**Table 1:** Genotypes of the selected 18 *M. tuberculosis* clinical isolates and the infection models used to characterise them.

	Genotype					Infection models^d^		
Spec no	LSP lineage^e^	Spoligotype^a^	ST number^a^	Octal code^b^	MIRU code^c^	Macrophage	Dendritic cell	Mouse
119	BJ	BEIJING	ST1	000000000003771	233325173533	+		
345	BJ	BEIJING LIKE	Unclassified	000000000000671	233325143535	+		
212	BJ	BEIJING	ST1	000000000003771	233325173533	+	+	+
649	BJ	BEIJING LIKE	ST269	000000000000771	233225173434	+		
374	BJ	BEIJING	ST1	000000000003771	232325173543	+	+	+
333	BJ	BEIJING	ST1	000000000003771	254225223532	+	+	+
411	BJ	BEIJING LIKE	ST941	000000000003751	223325173533	+		
346	IO	EA14_VNM	ST139	777777774413771	364225223533	+	+	+
232	IO	EA14_VNM	ST139	777777774413771	364225123533	+	+	+
372	IO	EA14_VNM	ST139	777777774413771	364225123533	+	+	+
281	IO	ZERO	ST405	777777000000011	264225124533	+		
367	IO	ZERO	ST405	777777000000011	364225222533	+		
293	EA	T1	ST53	777777777760771	222325143322	+	+	+
173	EA	H1	ST62	777777774020731	235323143423	+		
355	EA	T1	ST53	777777777760771	252325153322	+	+	+
318	EA	T1	ST53	777777777760771	215325143323	+	+	+
440	EA	H3	ST946	777777740020771	232324152323	+		
639	EA	H3	ST50	777777777720771	232325154323	+		

a Classified in accordance with international spoligotyping database [19].

b Octal code classification according to the recommendations of Dale et al [17].

c 12-loci MIRU using loci 2,4,10,16,20,23,24,26,27,31,39,40 [18],

d ‘+’ indicates the strain was tested in the model

e LSP lineage: BJ- East Asian/Beijing, IO-Indo-Oceanic and EA- Euro-American

### Characterisation in murine macrophages

We hypothesised that the different ability of *M. tuberculosis* lineages to cause disease is driven by their ability to skew the innate immune response. We therefore characterised the behaviour of the 18 selected strains in murine bone-marrow-derived macrophages. We compared their behaviour with H37Rv (the sequenced type-strain) and strain HN878, the previously characterised East Asian/Beijing strain associated with an anti-inflammatory phenotype (compared with H37Rv) in human macrophages [11].

**Figure 2:**
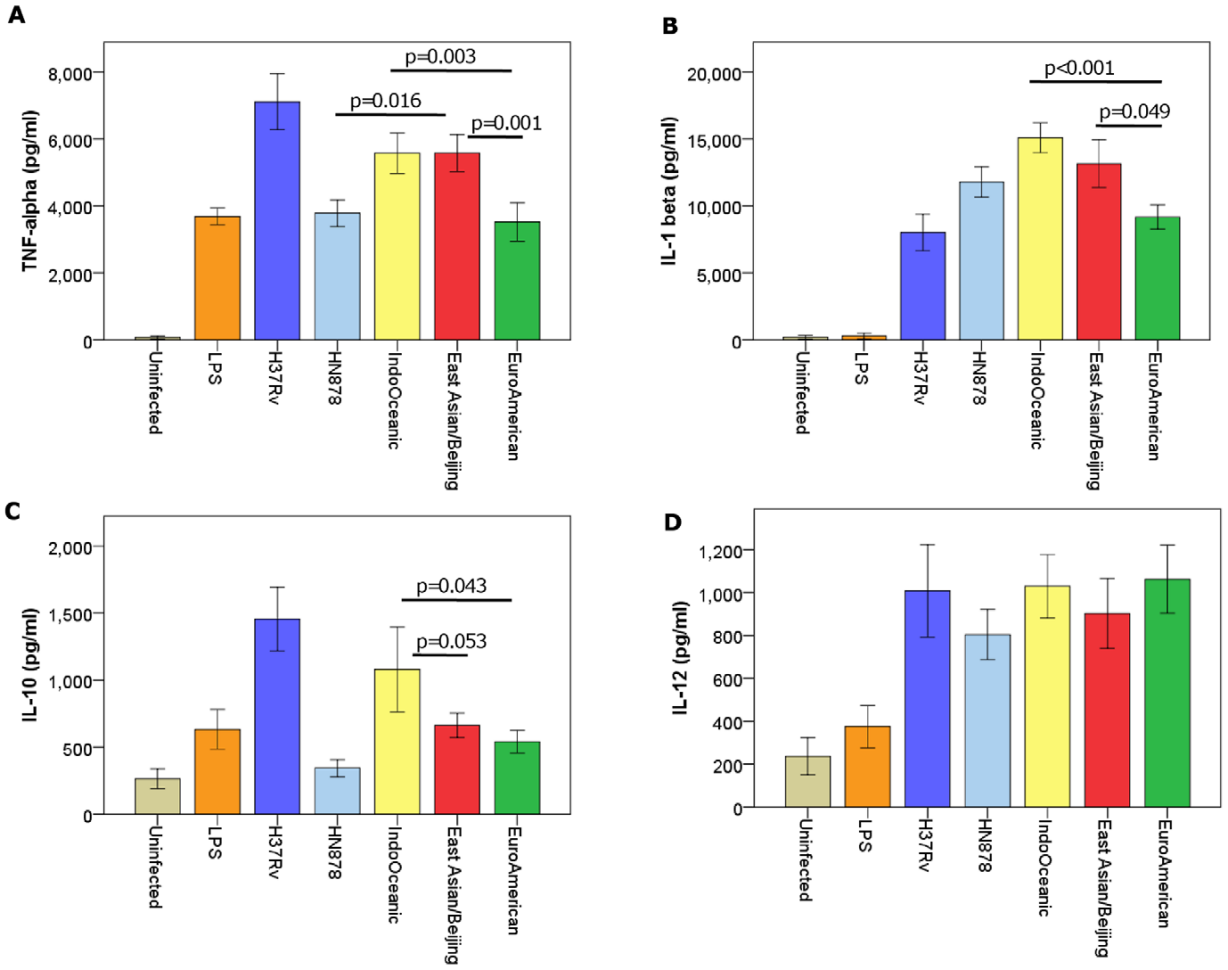
*M. tuberculosis* lineage influences cytokine expression from infected macrophages. Bone marrow derived macrophages were infected with 6 Euro-American, 7 East Asian/Beijing and 5 Indo-Oceanic strains at a M.O.I of 5, for 72 hours. Cytokine concentrations in the cell supernatants were determined using ELISA. TNF-α concentration at 24 hours (A), IL-1β at 48 hours (B), IL-10 levels at 48 hours (C) and IL-12p40 levels at 72 hours (D) are displayed. Beijing and Indo-Oceanic strains induced more pro-inflammatory TNF-α (Beijing, p = 0.001; Indo-Oceanic, p = 0.003) and IL-1β (Beijing, p = 0.049; Indo-Oceanic, p<0.001) than Euro-American strains. Data represents mean ± standard deviation (SD) of three independent experiments.

Macrophages were infected with each strain over 72 hours in triplicate, and on three independent occasions. Cell supernatants were drawn at 24, 48, and 72 hours and the concentrations of cytokines determined by ELISA. Strains of the Indo-Oceanic and East-Asian/Beijing lineage were associated with significantly higher concentrations of pro-inflammatory cytokines TNF-α (p = 0.003, p = 0.001, respectively) and IL-1β (p<0.001, p = 0.049, respectively) than those of the Euro-American lineage (figure 2). There was a trend for the Indo-Oceanic strains to induce higher concentrations of IL-10 than the East Asian/Beijing (p = 0.053) and Euro-American strains (p = 0.043). HN878 was associated with significantly lower concentrations of TNF-α (p<0.001) and IL-10 (p<0.001) than H37Rv, as previously reported [11], but also induced significantly less TNF-α than the other representatives of the East Asian/Beijing lineage (p = 0.016).

**Figure 3:**
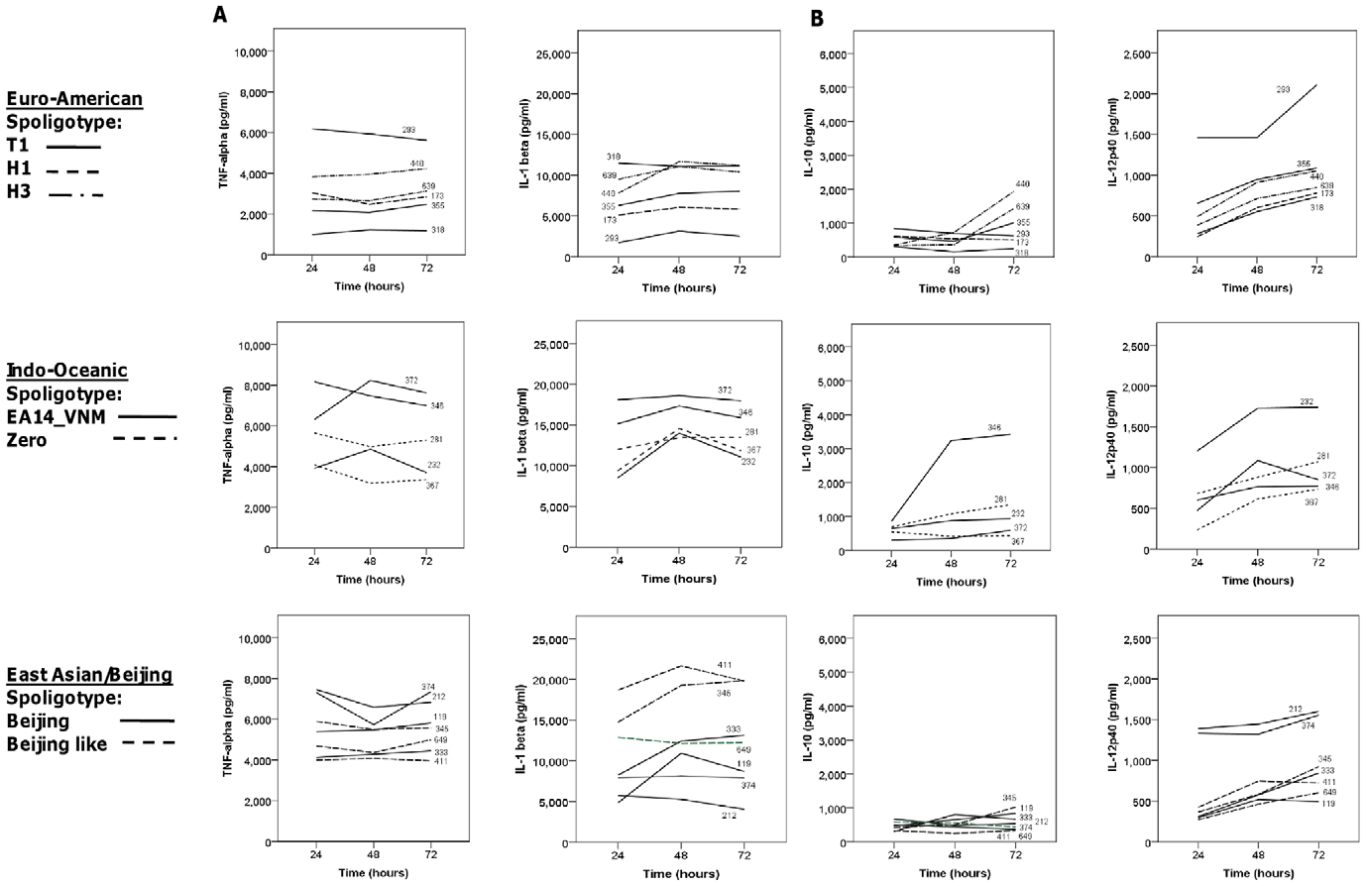
The cytokine profiles from macrophages infected with individual strains over 72 hours. Bone-marrow derived macrophages were infected with 6 Euro-American, 7 East Asian/Beijing and 5 Indo-Oceanic strains at a M.O.I of 5, for 72 hours. Cytokine concentrations for TNF-α and IL-1β (A) and IL-10 and IL-12p40 (B) were determined in the cell supernatants using ELISA. Lines represent the mean cytokine expression at 24, 48, and 72 hours of infection from three independent experiments, each performed in triplicate. The individual strains and their spoligotypes are labelled within the figure (see with table 1).

The contributions of individual strains within each lineage to the overall cytokine phenotype varied according to the lineage, the spoligotype subdivision, and cytokine measured (figure 3). There was greater heterogeneity of cytokine expression amongst the Indo-Oceanic and East Asian/Beijing strains than the Euro-American. In particular, strains of the Beijing-like spoligotype induced significantly more IL-1β than the classical Beijing spoligotype after 24, 48, and 72 hours of infection (p = 0.003, p = 0.014 and p = 0.011, respectively). One Indo-Oceanic strain (no. 346) induced much higher concentrations of IL-10 after 48/72 hours than any other strain, and was primarily responsible for the overall difference in IL-10 expression between the Indo-Oceanic and other lineages (figure 2). In addition, there was a tendency for the Indo-Oceanic ZERO spoligotype to induce less TNF-α and IL-1β than the EA14_VNM spoligotype after 24 and 48 hours of infection, although these differences did not achieve statistical significance (p>0.05). The spoligotype subdivisions within the Euro-American lineage did not significantly influence cytokine expression (figure 3).

### Characterisation in murine dendritic cells

**Figure 4:**
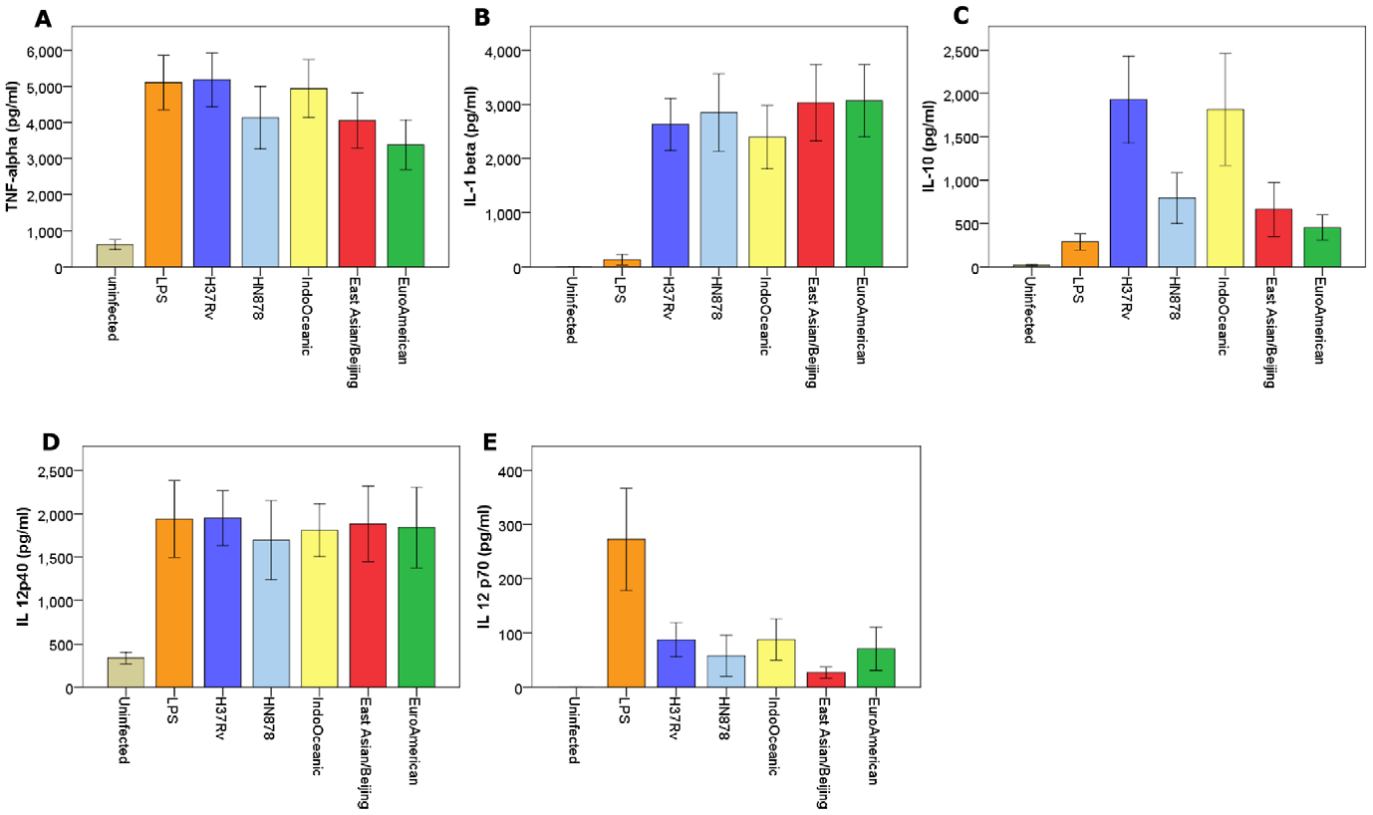
*M. tuberculosis* lineage does not significantly influence cytokine expression from infected dendritic cells. Bone-marrow-derived dendritic cells were infected with 3 Euro-American, 3 East Asian/Beijing and 3 Indo-Oceanic strains at a M.O.I of 5, for 72 hours. Cytokine concentrations in the cell supernatant were determined using ELISA. TNF-α concentration at 24 hours (A), IL-1β at 48 hours (B), IL-10 at 48 hours (C), IL-12p40 (D) and IL-12p70 levels (E) at 72 hours are displayed. There were no significant (p<0.05) differences in cytokine expression between any of the lineages, although there was a trend for the Indo-Oceanic strains to induce more IL-10 than the Euro-American strains. Data represents mean ± SD of three independent experiments.

**Figure 5:**
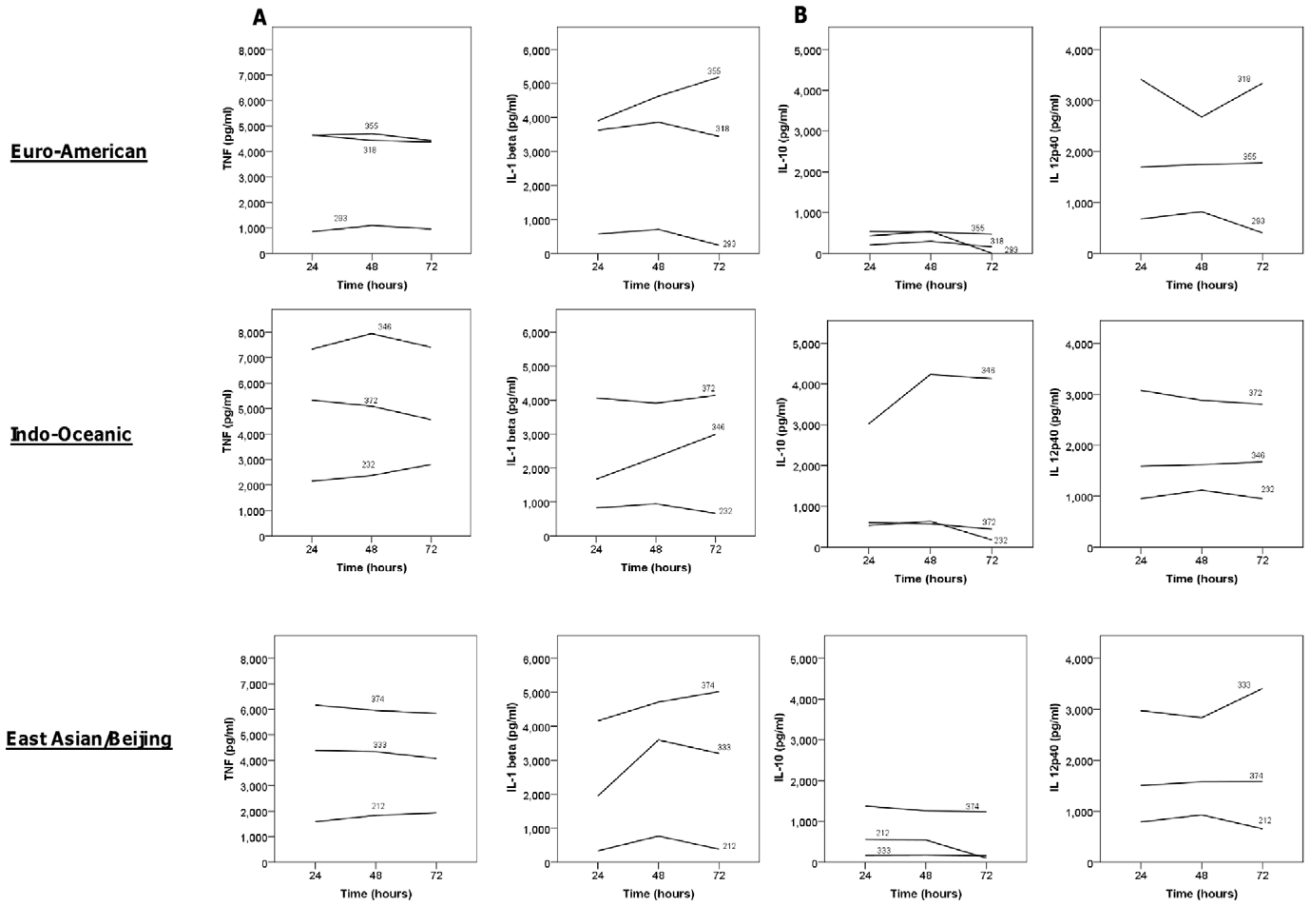
The cytokine profiles from dendritic cells infected with individual strains over 72 hours. Bone-marrow derived dendritic cells were infected with 3 Euro-American (all T1 spoligotype), 3 East Asian/Beijing (all Beijing spoligotype) and 3 Indo-Oceanic (all EA14_VNM spoligotype) strains at a M.O.I of 5, for 72 hours. Cytokine concentrations for TNF-α and IL-1β (A) and IL-10 and IL-12p40 (B) were determined in the cell supernatants using ELISA. Lines represent the mean cytokine expression of three independent experiments at 24, 48, and 72 hours of infection from 3 independent experiments, each performed in triplicate. The individual strains are labelled within the figure (see with table 1).

The innate immune response to *M. tuberculosis* may also be driven by interactions with dendritic cells [20]. To investigate whether the lineages induced similar cytokine profiles in macrophages and dendritic cells we infected bone-marrow derived dendritic cells with a subset of the clinical strains. Nine strains were selected: 3 East Asian/Beijing (all classical Beijing spoligotype), 3 Indo-Oceanic (all EA14_VNM spoligotype) and 3 Euro-American (all T1 spoligotype) (see table 1). Dendritic cells were infected with each strain over 72 hours in triplicate, and on three independent occasions. Cell supernatants were drawn at 24, 48, and 72 hours and the concentrations of cytokines determined by ELISA. Unlike the macrophage infections, there were no significant differences between the lineages in TNF-α or IL-1β production; although there was a similar trend for the Indo-Oceanic strains to induce higher concentrations of IL-10 than strains from the other two lineages (figure 4). There was, however, greater heterogeneity of cytokine responses between individual strains than observed for the macrophage infections (figure 5). In particular, Indo-Oceanic strain 346 induced significantly higher concentrations of IL-10 from both macrophages (figure 3) and dendritic cells (figure 5) than all the other strains tested. This strain was responsible for the trend, also observed in the macrophages, for the Indo-Oceanic lineage to induce higher concentrations of IL-10 than the other lineages. There were no significant lineage-specific differences in IL-12p40 or IL-12p70 expression.

### Characterisation in mice

The development of tuberculous meningitis in humans is dependent upon the extra-pulmonary dissemination of *M. tuberculosis* within the blood-stream [21] Extra-pulmonary dissemination is likely to occur early in infection, before the development of a protective T-cell mediated immune response [22]. We hypothesised that the ability of some lineages to cause more disseminated disease than others in humans would be associated with a greater capacity for haematogenous dissemination in a mouse model of infection. To test our hypothesis we used an intranasal infection model, as it has been shown previously, to be a useful model for assessing and unravelling pathways of mycobacterial dissemination [23].

**Figure 6:**
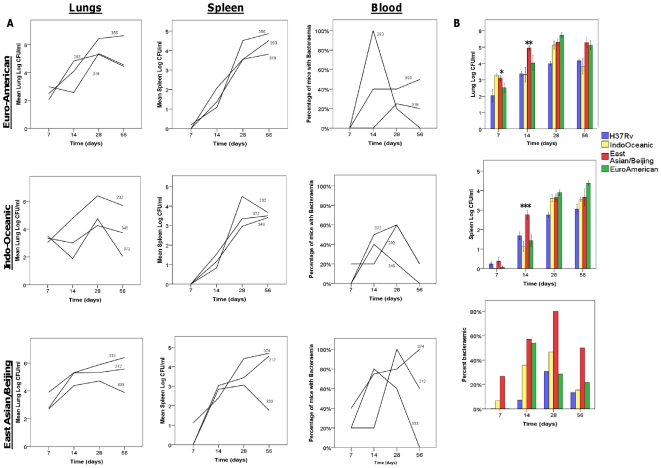
East Asian/Beijing lineage is associated with increased bacterial load in the lungs and spleen at early time points and bacteraemia. BALB/c mice were infected with 10^4^ CFU of H37Rv and three representative isolates of the Euro-American, East Asian/ Beijing and Indo-Oceanic lineages of *M. tuberculosis*. At the indicated time-points bacterial load was measured in the lungs, spleen, and blood. Mean CFU or the percentages of mice with bacteria cultured from blood (5 mice per group per time-point) are given for individual strains (A) (with the strain numbers indicated within each graph). The data are combined and presented by lineage in (B). Asterix (*) indicate significant (p<0.05) differences between lineages: * Indo-Oceanic and East Asian/Beijing > Euro-American (p = 0.01 and p = 0.08, respectively), ** East Asian/Beijing > Euro-American (p = 0.004), *** East Asian/Beijing > Euro-American (p = 0.002). Data represents mean ± 1 SD of three independent experiments, or percentage of 5 mice per group per time-point.

**Figure 7:**
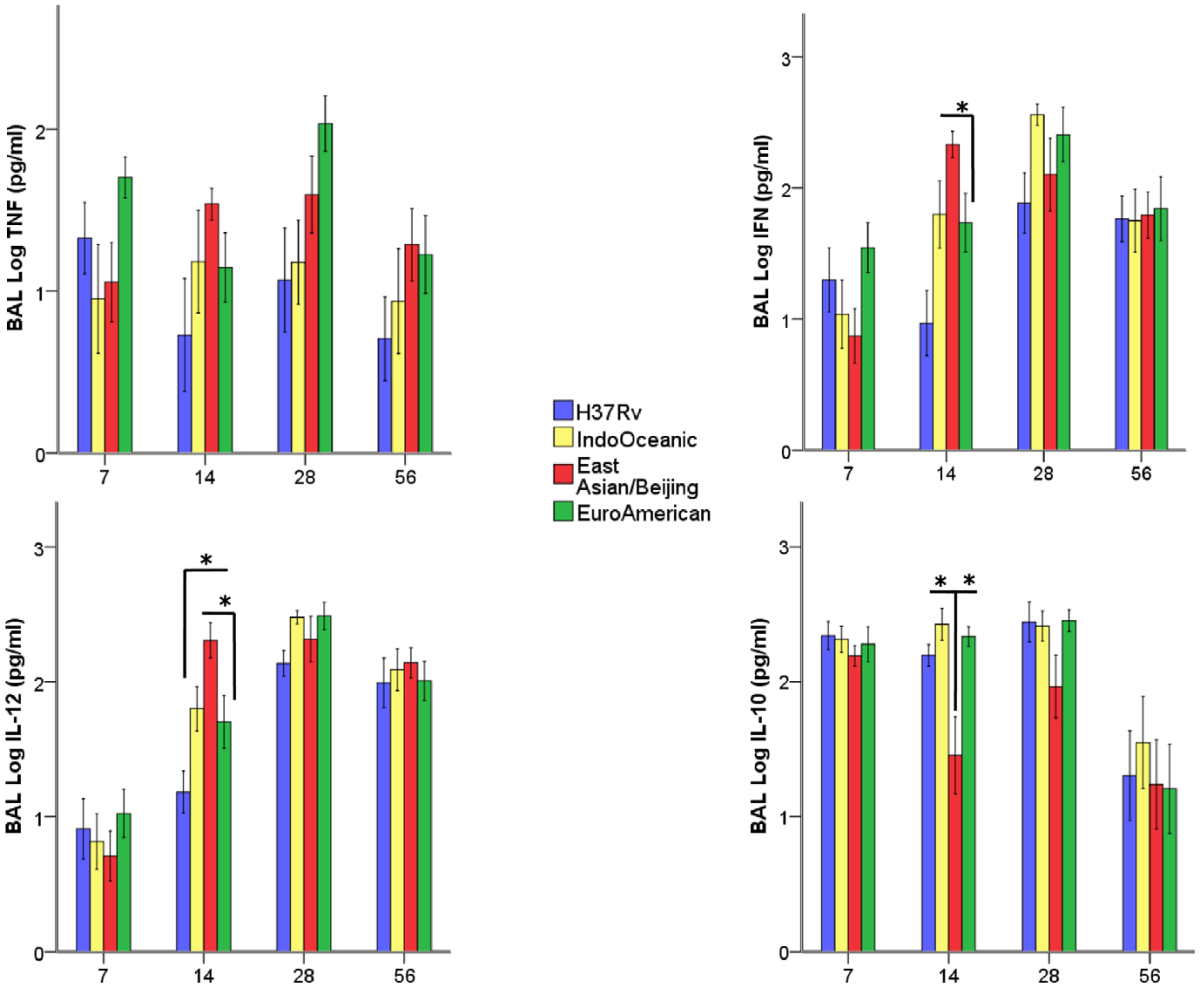
Pulmonary cytokine expression varies according to the infecting lineage of *M. tuberculosis.* Indicated cytokines were measured in the BAL using ELISA. Data are representative of three independent experiments, each with 5 mice per group. Bars represent mean +/- 1 SD. * signifies p-value of <0.05.

**Figure 8:**
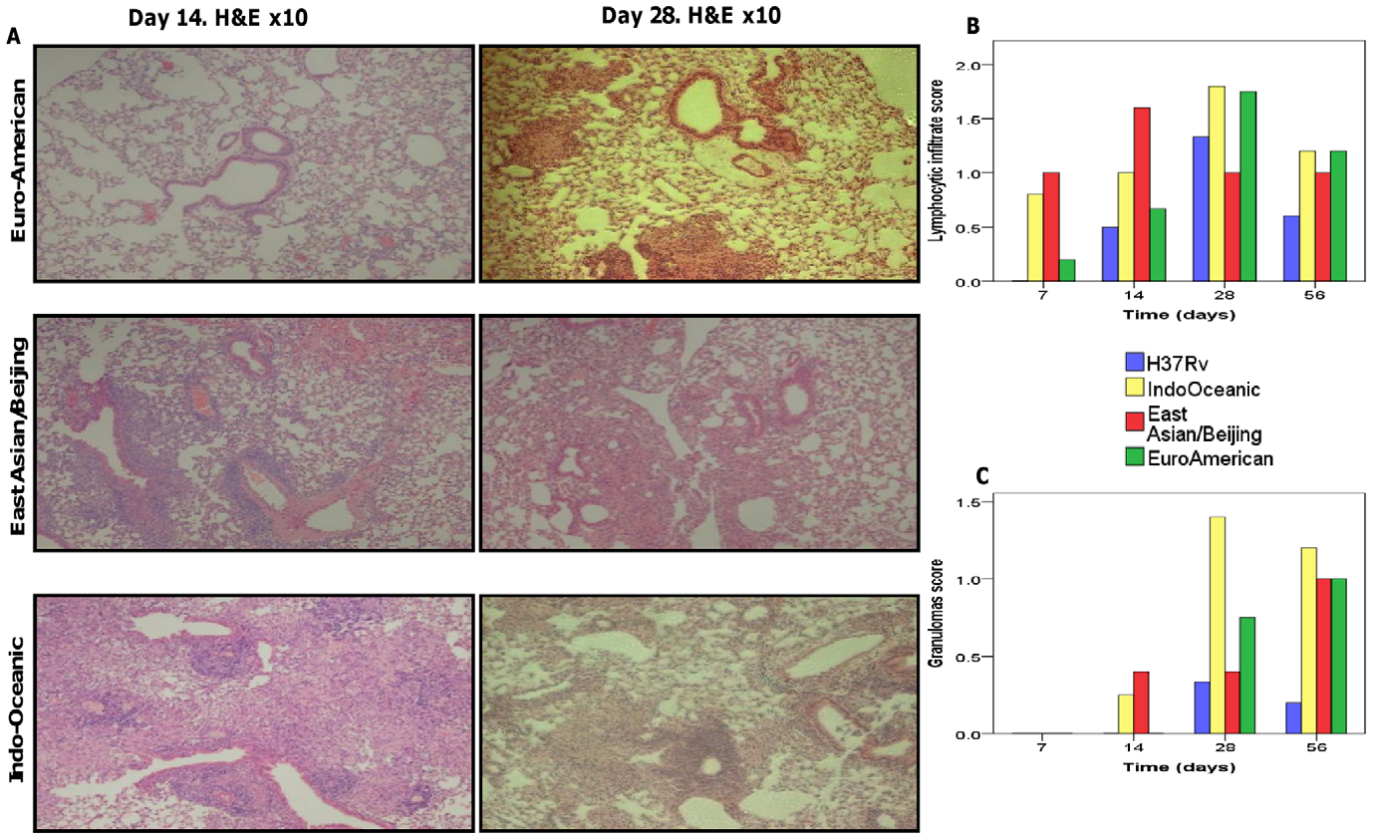
Histology of lungs infected with different lineages of *M. tuberculosis*. Sections of the lungs were stained with H&E stain. Representative lung sections for each *M. tuberculosis* infected group at day 14 and day 28 post-infection are displayed (A). Lung sections were scored (blind to the strain lineage) for lymphocytic infiltrates (B) and granulomas (C) and mean scores for each group presented. A score of 0 =  normal lung; a score of 2 =  moderate lymphocytic infiltrate/granuloma formation seen; a score of 3 =  extensive lymphocytic infiltrate/granuloma formation seen. The displayed histology images are from one experiment only.

Mice were infected intra-nasally with 10^4^ bacteria of representative strains from each lineage and H37Rv in three independent experiments. The nine strains tested were the same as those characterised in dendritic cells (table 1). The average infecting inoculums for each of the 3 lineages across the experiments was 1.6×10^4^ for the East Asian/Bejing, 5.9×10^3^ for the Indo-Oceanic, 4.1×10^4^ for the Euro-American and 1.2×10^4^ for H37Rv. Lung, spleen and blood bacterial colony forming units (CFU), broncho-alveolar lavage (BAL) cytokine concentration, and lung histology were compared between strains on days 7, 14, 28 and 56 of infection (figures 6, 7, 8). The East Asian/Beijing and Indo-Oceanic strains grew significantly faster in the lungs over the first 7 days, and by day 14 the East Asian/Beijing strains had achieved CFU concentrations in the lungs of 1 log greater than the other lineages (figure 6). The East Asian/Beijing strains caused a significantly greater proportion of mice to be bacteraemic over a longer time-period than the other lineages (figure 6).

Significant differences in BAL IL-12p40, interferon-γ, and IL-10 concentrations were observed between lineages (figure 7). At day 14 of infection, strains of the East Asian/Beijing lineage induced significantly higher concentrations of IL-12p40 and interferon-γ in BAL, and lower concentrations of IL-10, than strains of the other lineages. Strong correlations were observed between the dissemination of bacteria to blood and spleen and BAL concentrations of IL-12p40 and interferon-γ (table 2).

**Table 2:** Correlations between *M. tuberculosis* CFU in blood and spleen and inflammatory response in the lungs.

	Lung variable	Spearmans' correlation coefficient	P-value
**Blood CFU**	Lung CFU	0.321	<0.001
	Spleen CFU	0.235	0.004
	BAL IL-12p40	0.282	<0.001
	BAL IFN	0.161	0.046
**Spleen CFU**	Lung CFU	0.701	<0.001
	Blood CFU	0.235	0.004
	BAL IL-12p40	0.279	0.008
	BAL IFN	0.161	0.046

Univariable analysis showed the presence of bacteria in the blood was significantly associated with bacterial lineage, high lung and spleen CFU, and high concentrations of IL-12p40 and interferon-γ (table 3). After adjustment for CFU in the lung by multivariate logistic regression, the East Asian/Beijing strains were significantly more likely to be bacteraemic at any time-point, suggesting these strains may have a special ability to pass into the blood-stream, independent of their replication in the lung.

**Table 3:** The variables associated with the presence of *M. tuberculosis* in the blood of mice at any time-point following infection.

Univariate analysis^a^		Multivariate analysis b	
Variable	P-value	Odds ratio (95% confidence interval)	P-value
Lung CFU	<0.001		
Spleen CFU	<0.001		
Lineage:			
Euro-American		Reference	
Indo-Oceanic	0.001		
East Asian/Beijing		3.40 (1.44–8.03)	0.005
BAL IL-12p40	<0.001	1.003 (1.001–1.005)	0.024
BAL Interferon-γ	0.004		

a Mann Whitney U for continuous variables; Chi square for categorical

b Forward step-wise logistic regression. Spleen CFU was excluded from the model. Euro-American lineage was the reference.

Differences in pulmonary histopathology between the lineages were apparent during the first 14 days of infection only (figure 8). Lymphocytic infiltration was earlier and more extensive in the lungs of mice infected by Indo-Oceanic and East Asian/Beijing strains, and granulomas were only seen by day 14 of infection in mice infected with East Asian/Beijing or Indo-Oceanic strains. By day 56 of infection the differences in pulmonary pathology between the clinical strains were no longer observed, although H37Rv induced fewer granuloma and less lymphocytic infiltrate than the clinical isolates.

### Murine macrophage stimulations with whole lipid extracts from bacteria

**Figure 9:**
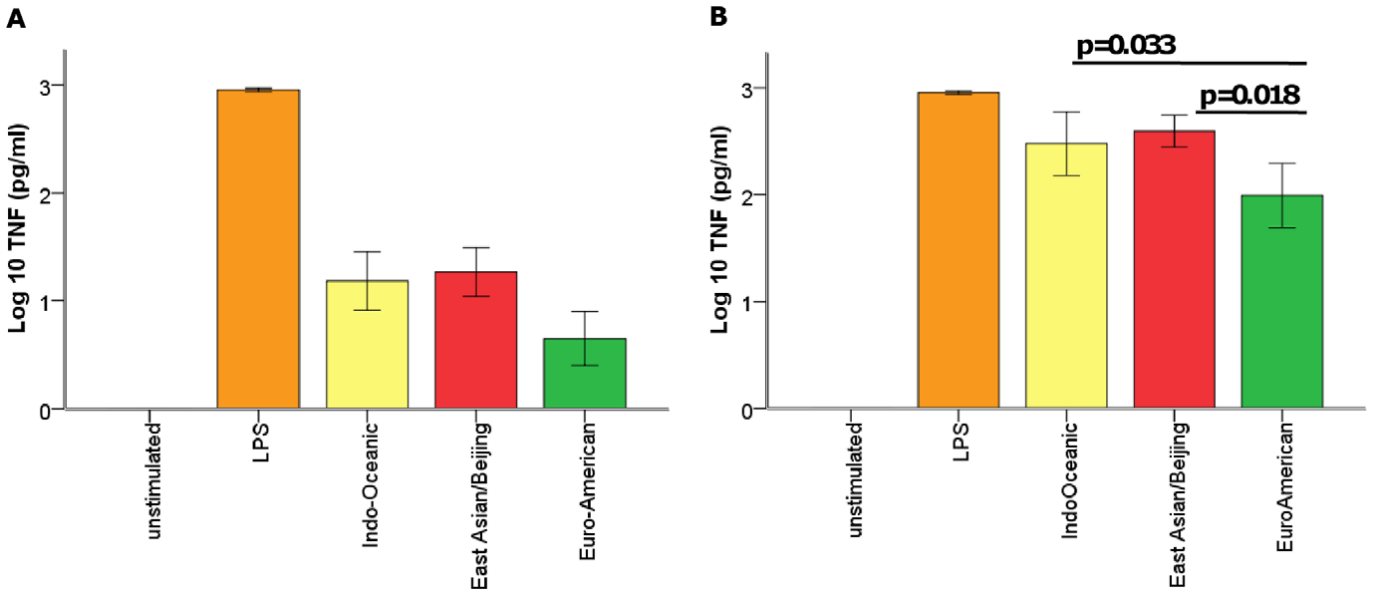
Total lipid extracts from East Asian/Beijing and Indo-Oceanic strains induce higher concentrations of TNF-α from macrophages than Euro-American extracts. Macrophages were incubated with 0.1 µg (A) and 1 µg (B) of total lipid extracts from *M. tuberculosis* strains from the different lineages. Culture supernatants were analysed by ELISA at 24 hours. Data represents means ±1SD of 6 Euro-American, 7 East Asian/Beijing and 5 Indo-Oceanic strains performed in triplicate.

To investigate whether bacterial cell-surface expressed lipids might be responsible for the difference in inflammatory phenotype observed following the infection of macrophages, we used extracted whole lipids from each of the strains to stimulate murine bone-marrow-derived macrophages. As observed for the macrophage infections, the East Asian/Beijing and Indo-Oceanic strain lipids (at concentration 0.1 µg and 1.0 µg) induced significantly higher concentrations of TNF-α than those from the Euro-American strains after 24 hours of stimulation (figure 9). In support of this observation, there was a correlation between the TNF-α induced by macrophage infection and the lipid stimulation at 24 hours for all strains (correlation coefficient 0.28, p = 0.061), although the correlation was strongest for the East Asian/Beijing strains (correlation coefficient 0.54 p = 0.022). The lipids did not induce lineage-specific differences in IL-10, IL-12p40, or IL-1β expression (data not shown). Indeed, the lipids induced very low concentrations of IL-1β (median 0 pg/ml for each lineage; range 0-1671 pg/ml for Euro-American strains, range 0–83 pg/ml for East Asian/ Beijing strains, range 0–899 pg/ml for Indo-Oceanic strains).

### Characterisation of the bacterial cell wall lipids

**Figure 10:**
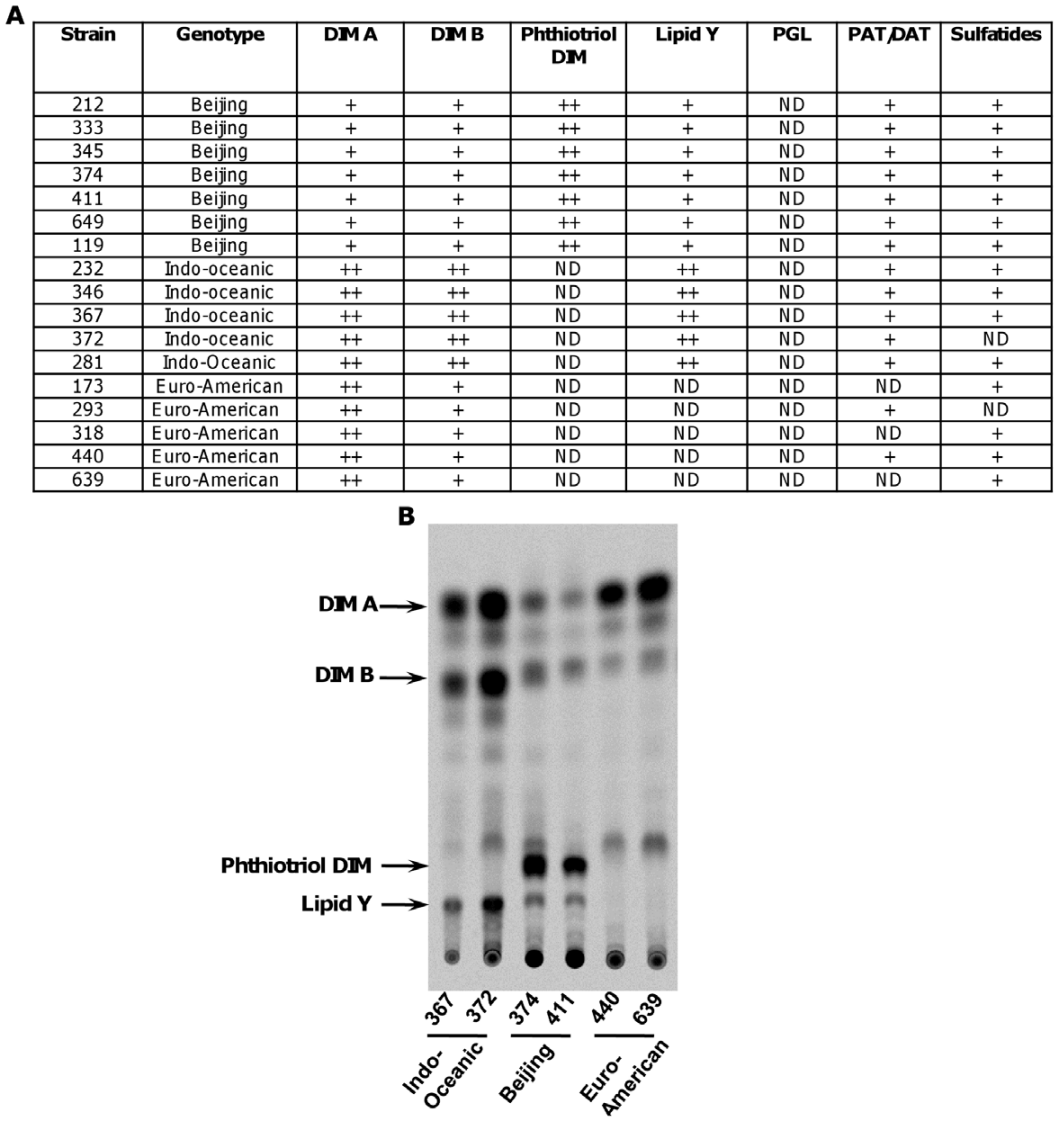
The cell envelope lipid profile varies according to *M. tuberculosis* lineage. The lipid profile of each strain was examined by thin layer chromatography (TLC) of the crude lipid extract using various solvents (A). TLC analysis of lipids from representative Indo-Oceanic, East-Asian/Beijing or Euro-American strains is displayed. Lipids were radiolabelled with ^14^C- propionate. The TLC was run in petroleum ether/diethylether (90∶10, v/v) (B).

With the aim of identifying individual lipids that may contribute to lineage-specific differences in inflammatory response, total lipid extracts were fractionated and compared by thin layer chromatography (TLC). We observed a distinct pattern of lineage-associated profiles (figure 10). We found variations in the content of sulfatides and other acylated trehalose derivatives but, since these varied between strains from the same lineage, we did not pursue them further. The most striking lineage-specific profiles were evident amongst lipid families related to phthiocerol dimycocerosate. Although all of the East Asian/Beijing and Indo-Oceanic strains have an intact gene for polyketide synthase 15/1 [24], [25], we were unable to detect any production of PGL, the lipid component implicated in immune subversion by HN878. As previously observed for laboratory strains H37Rv and Erdman, the profile of the Euro-American strains was characterised by large amount of phthiocerol dimycocerosate (DIM A) and smaller amounts of phthiodiolone dimycocerosate (DIM B). Also consistent with previous reports, East Asian/Beijing strains were characterized by the production of phthiotriol dimycocerosate. This is caused by a point mutation in *Rv2952* that encodes a methyltransferase. This mutation has a profound impact on the O-methyltransferase activity, and mutation complementation experiments confirmed the role of this mutation in the accumulation of phthiotriol dimycocerosate in Beijing strains [26]. We also observed a lineage-specific lipid profile for the Indo-Oceanic strains, comprising two spots migrating with DIM A and DIM B, and a novel unidentified spot labelled as ‘lipid Y’ in figure 10. To investigate the potential contribution of lipid Y to the pro-inflammatory phenotype observed for Indo-Oceanic strains, we undertook its structural and immunological analysis.

### Characterisation of lipid Y

**Figure 11:**
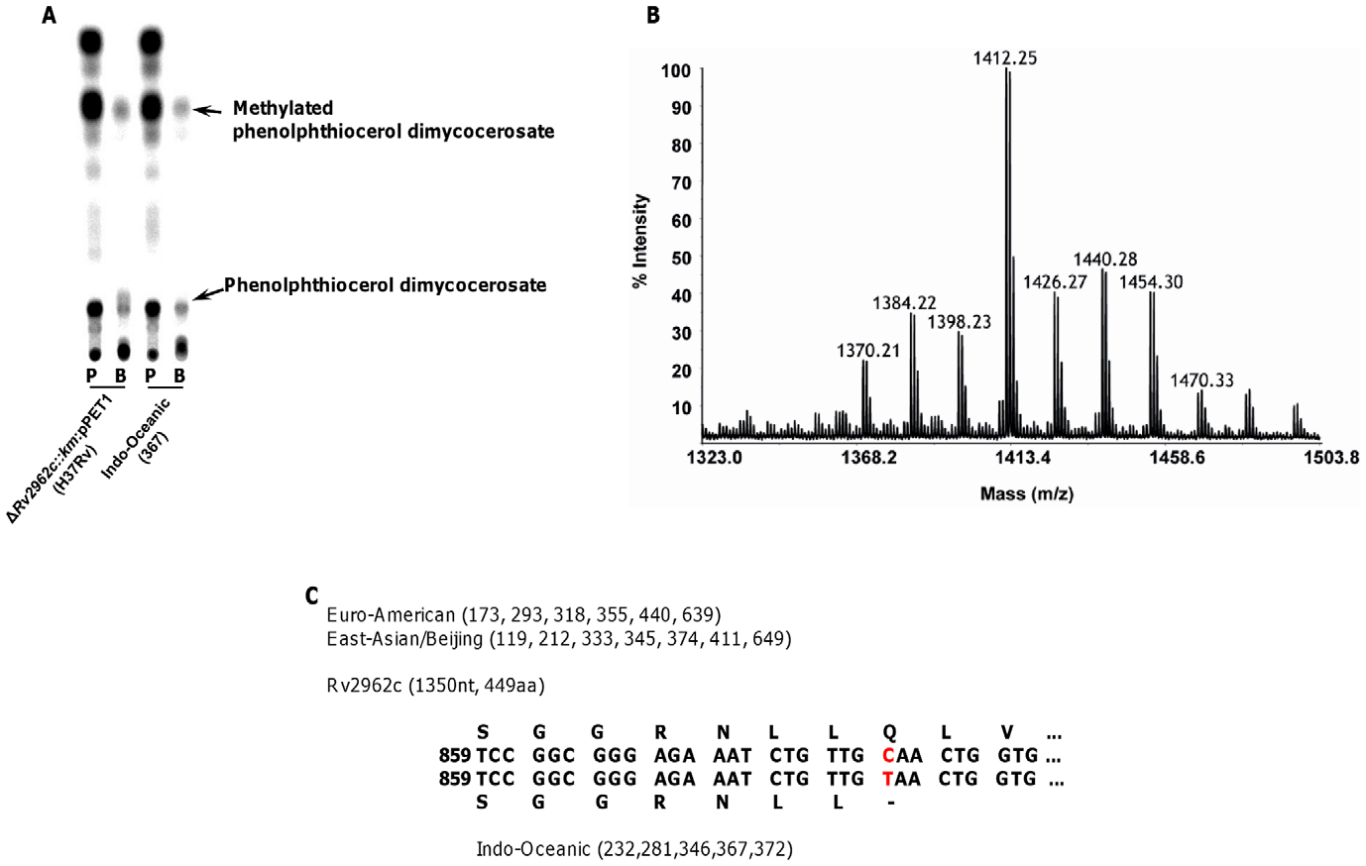
Lipid Y is a phenolphthiocerol dimycocerosate. One representative Indo-Oceanic strain (no. 367) and H37Rv *Rv2962c::km*: pPET1 (complemented *pks15/1*, disrupted *Rv2962c*) [27] were incubated with either ^14^C- propionate (lane ‘P’) or ^14^C-*p*-hydroxybenzoic acids (lane ‘B’) to label methyl-branched fatty acids (such as those found in PGL and DIM) or the phenol ring of PGL respectively. Thin layer chromatography revealed super-imposable spots (A). Lipid Y and lipids exhibiting *R*_f_ values similar to DIM B were then separated on a SepPak silica column and the enriched fractions analyzed by MALDI-TOF MS and ^1^H-NMR. The mass spectrum of lipid Y showed a series of pseudomolecular ion (*M* + Na^+^) peaks centered at *m/z* 1412 (B), identical to the ones obtained for phenolphthiocerol dimycocerosates purified from H37Rv Δ*Rv2962c::km*: pPET1. A SNP at position nt 880 of gene *Rv2962c* introduces a stop codon and a truncated open reading frame (C) in Indo-Oceanic strains.

In a previous study, investigating the role of a glycosyltransferase in the biosynthesis of *M. tuberculosis* phenolic glycolipids [27], it was shown that mutation of *Rv2962c* in an H37Rv-derived strain expressing a functional *pks15/1* gene, resulted in accumulation of two lipids: a phenolphthiocerol dimycocerosate and a methylated phenolphthiocerol dimycocerosate [27]. The lipid profile from H37Rv lacking *Rv2962c* but expressing *pks15/1* observed in the solvent system (petroleum ether/diethyl ether 9∶1) used to separate DIM, revealed three main spots: one containing DIM A, a second containing DIM B and methylated phenolphthiocerol dimycocerosate, and a third with a Rf very similar to lipid Y and phenolphthiocerol dimycocerosate. To get further insight into the nature of lipid Y, we compared lipid profiles generated after metabolic labelling of a representative Indo-Oceanic strain with a recombinant strain of H37Rv engineered to include an intact *pks15/1* but lacking *Rv2962c* (Δ*Rv2962c::km*: pPET1 [27]) (figure 11). Two compounds, ^14^C-propionate or ^14^C-p-hydroxybenzoic acids, were used to label methyl-branched fatty acids ( such as those found in PGL and DIM) or the phenol ring of PGL (found in PGL-like substances) respectively. Both strains produced identical profiles suggesting that Indo-Oceanic strains produced phenolphthiocerol dimycocerosate (lipid Y) and the associated methylated phenolphthiocerol dimycocerosate. This was confirmed by MALDI-TOF MS analysis of purified lipid Y and purified lipids exhibiting the same Rf as DIM B, showing a series of pseudomolecular ion (*M* + Na^+^) peaks centred at *m/z* 1412 (figure 11) and at *m/z* 1426 (data not shown), characteristic of phenolphthiocerol dimycocerosates and methylated phenolphthiocerol dimycocerosates respectively. In addition, ^1^H-NMR analyses of purified lipid Y revealed all the proton signal resonances expected for phenolphthiocerol dimycocerosates (data not shown). Thus, the lineage-specific lipid profile of Indo-Oceanic strains is generated by production of phenolphthiocerol dimycocerosate (lipid Y) and methylated phenolphthiocerol dimycocerosate (co-migrating with DIM B in figure 11).

Examination of the sequence of *Rv2962c* orthologs from Indo-Oceanic, East Asian/Beijing and Euro-American isolates revealed a single nucleotide polymorphism (SNP) at position nt 880. This SNP introduces a stop codon and leads to a truncated *Rv2962c* open reading frame in Indo-Oceanic strains (figure 11C). This SNP provides an explanation for the accumulation of phenolphthiocerol dimycocerosate in strains of this lineage.

### Role of phenolphthiocerol dimycocerosate (lipid Y) in inflammatory phenotype

**Figure 12:**
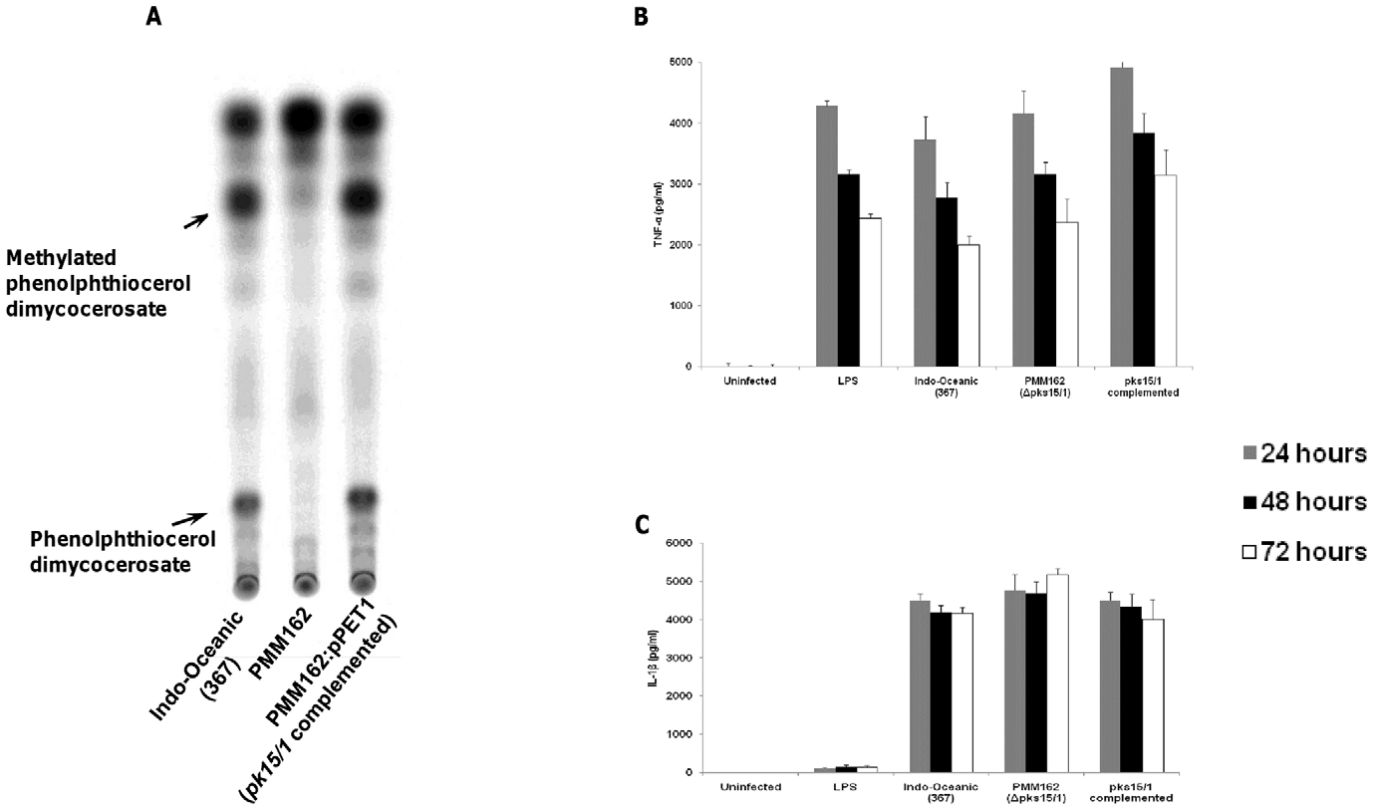
Phenolphthiocerol dimycocerosate does not influence TNF-α and IL-1β production from macrophages. TLC analysis of lipids from Indo-Oceanic strain 367, its isogenic *pks15/1::km* mutant (PMM162) and *pks15/1* complemented strain (PMM162:pPET1). Lipids were radiolabelled with ^14^C- propionate. The TLC was run in petroleum ether/diethylether (90:10, v/v) (A). Bone marrow derived macrophages were infected at an M.O.I of 5 for 72 hours with a representative Indo-Oceanic strain (no. 367) , mutant (PMM162) strain incapable of making lipid Y (*pks15/1* knock-out mutant) and the complemented strain . Cytokine concentrations in the cell supernatants were determined using ELISA. TNF-α (B) and IL-1β concentrations (C) are shown across the 3 time-points studied. Data represents mean ± standard deviation (SD) of three independent experiments.

Finally, to determine the influence of phenolphthiocerol dimycocerosate (lipid Y) production on inflammatory phenotype we compared the behaviour of a wild-type Indo-Oceanic strain (no. 367 – see table 1), the same strain with *pks15/1* mutated (PMM162), which cannot produce phenolphthiocerol dimycocerosate and the *pks15/1* complemented strain (figure 12A). Following infection of murine bone-marrow-derived macrophages we found the mutated strain induced similar levels of TNF-α as the wild-type and complemented strains (figure 12B). The mutant did not induce significant differences in IL-1β (figure 12C), IL-12p40, or IL-10 expression from macrophages (data not shown).

## Discussion

One of the most intriguing observations following *M. tuberculosis* infection is the diversity of possible outcomes. Various host factors – age, malnutrition and co-infection with HIV, for example - have a well-defined influence on the development of tuberculosis [28], but the role of bacterial genetic variation on the development of disease is less clear. To address this question we first performed a case-control study in southern Vietnam and found a significant association between the development of meningitis and strains of the East Asian/Beijing or Indo-Oceanic lineages rather than the Euro-American lineage [15]. In this study we used meningitis as a model of disseminated disease and investigated the basis for this clinical association by using different models of infection to investigate the host-pathogen interactions.

First, we found strains of the East Asian/Beijing and Indo-Oceanic lineage induced significantly higher concentrations of TNF-α and IL-1β from bone-marrow-derived macrophages than the Euro-American strains. Others have reported East Asian/Beijing strains to be associated with an anti-inflammatory phenotype (with reduced TNF-α expression) [11], [29], [30], [31], but the strength of this finding depends upon the type of macrophages infected and the comparator strains used in the experiments. These investigators used either human peripheral blood monocyte-derived macrophages [11], [29] or immortalised human THP-1 macrophages [30], [31], and compared cytokine induction with Beijing strains and the laboratory strain H37Rv. Others have used murine bone-marrow derived macrophages and conversely found Beijing strains induced significantly higher concentrations of TNF-α and IL-1β than H37Rv [32]. Macrophage type may, therefore, influence cytokine expression and future experiments will involve characterisation of these strains in human macrophages to test the reproducibility of these phenotypes. There is also increasing evidence that sequential *in vitro* culture can induce important genetic and phenotypic inter-laboratory differences between strains of H37Rv [33], [34], [35]. H37Rv was isolated from a patient with tuberculosis more than 100 years ago and its subsequent serial use in laboratories worldwide may have compromised its use as a comparator strain between investigations and centres. Indeed, we found East Asian/Beijing strains (including HN878) induced substantially lower concentrations of TNF-α compared to H37Rv, and although this agrees with previous studies, we consider it less relevant to the aims of this study than the significant differences observed between the clinical strains. The macrophage phenotypes observed with the East Asian/Beijing strains in our study vary from a study reported by Portvein et al [14]. Commonly, the East Asian/Beijing lineage is considered a homogenous lineage but a number of subdivisions exist, each of which are characterised by different RD regions [36]. Although the strains used in both studies belong to the same lineage, it is possible they are from different genetic sub-divisions which may account for phenotypic differences observed.

As others have observed [5], the East Asian/Beijing strains grew faster in the lungs and disseminated to blood and spleen more efficiently than strains from the other lineages. Strains of the Indo-Oceanic lineage shared some of these properties, in particular the early rapid replication in lungs and the ability to cause bacteraemia through all the time-points studied. In addition, pulmonary granulomas were seen by day 14 in East Asian/Beijing and Indo-Oceanic strain infections, but took longer to form in mice infected with Euro-American strains or H37Rv. These differences may be explained by the pro-inflammatory phenotype of East Asian/Beijing and Indo-Oceanic strains observed in macrophages: enhanced expression of TNF-α and IL-1β early in infection might drive pulmonary granuloma formation through enhanced chemokine secretion, and ultimately promote bacterial dissemination. Granulomas have conventionally been considered to be structures that contain and prevent the spread of mycobacteria. Recent studies in embryonic zebrafish have challenged this assumption by demonstrating that macrophages within granulomas are responsible for mycobacterial dissemination during the early stage of infection [37], [38]. Applying this model, *M. tuberculosis* strains that promote early granuloma formation through enhanced expression of TNF-α may paradoxically disseminate earlier and more effectively. However, a recent study in a non-human primate model reported that neutralisation of TNF-α enhanced bacterial dissemination and increased bacterial burden in the lungs [39]. From the literature, the functionality of TNF-α seems to vary depending on the model and time points assayed during the experiments [40]. Since TNF-α is a cytokine with multiple roles, it is challenging to assign a single role to this cytokine in the pathogenesis of *M. tuberculosis* infection.

Rapid bacterial dissemination may also benefit the host by enhancing antigen delivery and presentation at regional lymph nodes [40]. Indeed, the East Asian/Beijing strains induced earlier and higher concentrations of IL-12p40 and interferon-γ in BAL. Elevated TNF-α and IL-1β was not observed in the BAL of mice infected with East Asian/Beijing or Indo-Oceanic strains, but this may be explained by the contribution of other cells (e.g. neutrophils) and the adaptive immune response at the time-points BAL were taken (7– 56 days post- infection). The strong correlations observed between IL-12p40 and interferon-γ in BAL, and bacterial growth in blood and spleen, suggest *M. tuberculosis* strains more able to disseminate may induce an earlier T-cell mediated protective immune response. Surprisingly, however, early pulmonary granuloma formation and greater expression of IL-12p40 and interferon-γ was not associated with subsequent control of the infection and arrest of the bacteraemia: half the mice infected with East Asian/Beijing strains were still bacteraemic by day 56 of infection. The failure to control the infection may reflect continued immune subversion by the bacteria. For example, the East Asian/Beijing strains induced significantly less pulmonary IL-10 on days 14 and 28 of infection than the other strains, which may enhance on-going immune-pathology and dissemination.

*M. tuberculosis* has a complex, lipid rich cell wall which is responsible for many of its unique properties (acid-fast staining and resistance to many antibiotics, for example) and it has long been considered important in the pathogenesis of tuberculosis [41], [42]. Having demonstrated significant phenotypic differences between the major lineages in macrophages and mice we hypothesised these differences may be driven by cell surface-expressed lipids. We found whole lipid extracts from the clinical strains induced a similar pattern of TNF-α expression from macrophages to the live infections, strongly suggesting these molecules influence the innate immune response to infection with different *M. tuberculosis* strains. Others have reported a similar correlation between lipid- and live infection-induced TNF-α expression [43], [44]. Unlike the live infections, however, lipids failed to induce IL-1β from macrophages. IL-1β expression is dependent on the cleavage of its precursor pro-IL-1β, commonly by caspase-1, which in turn is activated by protein platforms in the cytosol called inflammasomes [45]. *M. tuberculosis* induces IL-1β expression from macrophages through pathways involving TLR2/TLR6 and intracellular NOD2 receptors [46]. Lipid extracts (and LPS) may not induce IL-1β because they fail to enter the cytosol and activate NOD2. The influence of *M. tuberculosis* lipids on IL-1β expression may, therefore, be better investigated by live bacterial infections and our current findings do not exclude a significant role for these molecules in differential IL-1β expression.

Bacterial cell wall lipid composition was highly conserved within the lineages, particularly amongst the lipid families related to phthiocerol dimycocerosate. This suggests these molecules may be important in lineage-related phenotypic differences. The East Asian/Beijing strains all produced phthiotriol dimycocerosate, but recent investigations did not find a specific role for this molecule in virulence [26]. More intriguing was a unique lipid produced by the Indo-Oceanic strains alone, which we subsequently showed to be a phenolphthiocerol dimycocerosate. Nearly 40 years ago, Goren and others described the same lipid being produced by clinical *M. tuberculosis* strains from southern India, but not by strains isolated in the UK [47]. The Indian strains were shown to be less virulent than the UK strains in guinea pigs [3] and because of the strong association between the presence of the lipid and impaired virulence, it was named ‘attenuation indicator lipid’ [47]. When we examined the inflammatory phenotype of an Indo-Oceanic strain unable to produce phenolphthiocerol dimycocerosate, through mutation of *pks15/1*, we found it induced similar concentrations of TNF-α compared to the wild-type. The significance of this finding is difficult to determine, but it seems that although the phenolglycolipid in the East-Asian isolate, HN878 is able to suppress the release of cytokines from macrophages [12] the related phenolphthiocerol dimycocerosate does not modulate cytokine responses from macrophages. This would suggest that the saccharidic domain, absent in lipid Y, plays a major role in this activity. Further studies are warranted to address the reasons why related lipids can generate diverse phenotypes with respect to the host innate immune response. On-going investigations in mice infected with Indo-Oceanic strains with phenolphthiocerol dimycocerosate synthesis disrupted will determine whether or not this lipid has a role in the virulence of Indo-Oceanic strains.

In conclusion, the *M. tuberculosis* lineages more likely to cause disseminated tuberculosis with meningitis in southern Vietnam are associated with a pro-inflammatory phenotype in macrophages. These lineages may subvert the host innate immune response to increase their survival and dissemination within the host. Bacteraemia is a critical step in the extra-pulmonary dissemination of *M. tuberculosis* and essential for the development of meningitis [40]. We found strains most strongly associated with the development of tuberculous meningitis in humans caused early and prolonged bacteraemia in mice. Finally, we have demonstrated for the first time, a highly conserved lipid profile within the strains of each lineage. Our findings suggest that lineage of *M. tuberculosis* may influence the early interactions with the host and may ultimately determine the clinical consequences of infection.

## Materials and Methods

### Ethics statement

All animal experimentation was performed under licence PPL 70/7160 issued by the UK Home Office according to the Animals (Scientific Procedures) Act 1986. The mice were obtained from Charles River Ltd, UK.

### Bacterial strains and culture conditions

*M. tuberculosis* strains were cultured in Middlebrook 7H9 liquid media supplemented with 0.2% glycerol, 0.05% Tween 80 and 10% oleic acid-albumin-dextrose-catalase (OADC), or in Sauton's media for lipid extraction. For growth on solid media, Middlebrook 7H10 plates were supplemented with 0.5% glycerol and 10% OADC. All the assays described below were performed blind to the infecting strain and its lineage. All the isolates were susceptible to all first-line anti-tuberculosis drugs.

### Isolation of mouse bone-marrow derived macrophages

Bone-marrow cells were flushed from the femur and tibia of 8 to 10 week old female BALB/c mice and differentiated into macrophages for 7 days in RPMI 1640 (Lonza) supplemented with 1 mM sodium pyruvate (Lonza), 2 mM L-glutamine (Lonza), 0.05 M 2-mercaptoethanol (Gibco), 10% heat-inactivated fetal bovine serum (Biosera) and 20% L-cell conditioned media. On day 4, cells were fed with an additional 10 ml of media. After 7 days in culture, cells were washed with phosphate-buffered saline (PBS) and seeded into 24-well plates at 5×10^5^ cells/well.

### Isolation of mouse bone-marrow derived dendritic cells

Bone marrow cells were flushed from the femur and tibia of 8 to 10 week old female BALB/c mice, and cells were seeded into 10 cm Petri dishes in RPMI 1640 supplemented with 1 mM sodium pyruvate, 2 mM L-glutamine, 0.05 M 2-mercaptoethanol, 5% heat-inactivated fetal bovine serum and 10 ng/ml of recombinant murine granulocyte-macrophage colony-stimulating factor (R&Dsystems). On days 2 and 4, the cells were fed by changing 75% of the media and non-adherent cells were harvested on day 6. Prior to infection with *M. tuberculosis*, cells were seeded into 48-well plates at 2×10^5^ cells/well.

### Lipid extraction and analysis

Each *M. tuberculosis* isolate was grown in 125 ml cultures in Sauton's medium without agitation in order to get a biofilm and high amount of material. Lipids were extracted by a first incubation in CHCl_3_/CH_3_OH (1∶2, v/v) for two days at room temperature and then in CHCl_3_/CH_3_OH (2∶1, v/v). The two organic phases were pooled, washed twice with water and dried to get crude lipid extracts. The lipid profile were compared by spotting equivalent amounts of crude extracts (resuspended in CHCl_3_ at a final concentration of 20 mg.ml^−1^) on TLC plates, which were then run in various solvent systems (CHCl_3_/CH_3_OH 95∶5 (v/v) for PGL; petroleum ether/diethyl ether 9∶1 (v/v) for DIM; CHCl_3_/CH_3_OH/H_2_O 60∶16∶2 (v/v) for DAT and SL; CHCl_3_/CH_3_OH 99∶1 (v/v) for PAT). Lipids were visualized by spraying the plates with 10% phosphomolybdic acid in ethanol, and glycolipids with a 0.2% anthrone solution in concentrated H_2_SO_4_, followed by heating. For structural analysis, lipids were fractionated by chromatography on a SepPak silica column. Increasing concentrations of diethyl ether in petroleum ether were used as eluents to obtain fractions enriched in the various lipids of interest.

For the metabolic labelling experiments, each strain was cultured (8 mL) to exponential growth phase and labeled by incubation with 0.625 µCi.ml^−1^ [1-^14^C] propionate (specific activity of 54 Ci.mol^−1^) or 0.875 µCi.ml^−1^ [1-^14^C] *p-*hydroxybenzoic acid (specific activity of 58 Ci.mol^−1^) for 24 hours. Lipids were extracted as described before [26]. The TLC plates were run in petroleum ether/diethyl ether (90∶10, v/v) and labeled lipids were visualized with a Typhoon PhosphorImager (Amersham Biosciences).

To screen for the presence of the SNP in Rv2962c, primers F1(5′-CTGCCCTCCACCATATACC-3′), R-2(5′-CTCTGCCCCACCAACAGTC-3′) and M1(5′-ACGGCCTCCATATTCAAGTG-3′) were used to amplify a 1.35 kb sequence from each of the Indo-Oceanic isolates. Following PCR amplification, the presence of the SNP was confirmed by DNA sequencing on an Applied Biosystems 3730 automated capillary DNA sequencer. The sequences have been deposited with GenBank (accession number JF925156).

### Lipid structural analysis

Purified molecules were analyzed by matrix-assisted laser desorption-ionization time-of-flight (MALDI-TOF) mass spectrometry, as previously described [48]. Spectra were acquired in reflectron mode, with an Applied Biosystems 4700 Analyzer mass spectrometer (Applied Biosystems, Framingham, USA) equipped with an Nd:YAG laser (wavelength 355 nm; pulse <500 ps; repetition rate 200 Hz). A total of 2500 shots were accumulated in positive ion mode and mass spectrometry data were acquired with default calibration. Nuclear magnetic resonance (NMR) spectroscopy experiments were carried out at 295°K on a Bruker AVANCE spectrometer operating at 600,13 MHz with a 5-mm triple resonance TCI ^1^H ^13^C ^15^N pulsed field z-gradient cryoprobe. Samples were dissolved in 99.9 % CDCl_3_. Chemical shifts are expressed in ppm using the chloroform signal as an internal reference (7.23 ppm).

### Cellular infection assays

Macrophages and dendritic cells were infected with *M. tuberculosis* from mid-log phase cultures at a multiplicity of infection (MOI) of 5. Control wells were stimulated with 50 ng/ml of lipopolysaccharide (LPS) from *Escherichia coli* (Sigma Aldrich). Supernatants were removed at 24, 48 and 72 h post infection and filtered using 0.22 µm filters (Millipore) before removing from the BSL3 lab. Concentration of cytokines in the supernatants was determined using an enzyme-linked immunosorbent assay (ELISA) according to the manufacturer's instructions (E-bioscience). Each *M. tuberculosis* strain was assayed in triplicates in three independent experiments. Macrophages were also stimulated using total lipid extracts. All the lipid extracts were tested for endotoxin using the limulus amebocyte assay QCL-1000 (Cambrex) and was below the detection level of the kit.

Lipid extracts were suspended at a concentration of 1 mg/ml in hexane/isopropanol and incubated for 5 minutes in a 600C bath sonicator. This step was repeated twice. The lipid solution was layered onto 24 well plates and the solvent was allowed to evaporate. Control wells were layered with hexane/isopropanol without lipid extracts. Macrophages were seeded on lipid coated plates at a concentration of 5×10^5^ cells/well and supernatants were collected at 3, 6 and 24 hours post-stimulation for analysis using ELISA. All the experiments were done blind to the bacterial lineage of each strain and in triplicate wells with macrophages obtained from three individual mice.

### Mouse infections

Female BALB/c mice (8–10 week old) were maintained in the BSL3 facility according to institutional protocols. Mice were infected with representative isolates of the Euro-American, Indo-Oceanic, and East Asian/Beijing lineages and H37Rv. Each mouse was infected with 1×10^4^ bacteria via the intranasal route. At the indicated time points post infection, bacterial burden was determined in the broncho-alveolar lavage (BAL), blood, spleen and lungs. Aseptically removed lungs and spleen were homogenised in PBS containing 0.05% Tween 80, and serial dilutions of the organ homogenates were plated. The number of colony forming units (CFU) was enumerated 21 days later. The upper left lobe of the lung was fixed in 10% buffered formalin for histopathological analysis. Paraffin embedded sections were stained with hematoxylin and eosin or Ziehl-Neelsen stains. Lung sections were scored (blind to the strain lineage) for lymphocytic infiltrates and granulomas and mean scores for each group calculated. A score of 0 =  normal lung; a score of 2 =  moderate lymphocytic infiltrate/granuloma formation seen; a score of 3 =  extensive lymphocytic infiltrate/granuloma formation seen.

All experiments were done blind to the lineage of the infecting strain.

### Construction of the Indo-Oceanic 367 *pks15/1* mutant and complemented strain

A fragment containing part of the *pks15/1* gene disrupted by the kanamycin (km) cassette was obtained from plasmid pCG134 [24] on a PmeI fragment and inserted within the XbaI-SpeI site (made blunt) of cosmid vector pYUB854. The resulting cosmid was cut with *Pac*I, ligated with the mycobacteriophage phAE87 and encapsidated *in vitro* using the Gigapack III XL kit (Stratagene). The *in vitro*-generated phage particles were used to infect *E. coli* HB101 and transfectants were selected on LB plates containing Km. A recombinant phagemid, named phWM14, containing the disrupted gene construct was selected and transferred by electroporation in *M. smegmatis* and phage particles were prepared as described previously [49]. These particles were then used to infect *M. tuberculosis* IO367 and allelic exchange mutants were selected by PCR analysis as described previously [24]. One clone, named PMM162 (Δ*pks15/1::km*), gave the pattern corresponding to allelic exchange and was retained for further analysis. The *pks 15:/1* complemented strain was produced by transferring plasmid pPET1 carrying a functional *pks15/1* gene into PMM162. Transformants were selected on plates containing kanamycin and hygromycin. One clone, named PMM162:pPET1, was retained for further analysis. Production of lipid Y in the complemented strain, PMM162:pPET1, was confirmed by lipid extraction, metabolic labelling with propionate and TLC analysis as described before [24].

### Statistical analysis

The data from each experiment were combined and analysed together by lineage. Continuous variables (e.g. cytokine concentration) were compared between strains by the Mann-Whitney U test and categorical variables were compared by the chi-square test or Fishers exact test. Correlations between continuous variables were calculated using Spearman's correlation. Multivariable analyses were performed using forward step-wise logistic regression.
